# Patent foramen ovale closure by using transesophageal echocardiography for cryptogenic stroke: single center experience in 132 consecutive patients

**DOI:** 10.1186/s13019-020-1042-4

**Published:** 2020-01-09

**Authors:** Yangyang Han, Xiquan Zhang, Fengwei Zhang

**Affiliations:** 1 Department of Cardiovascular Surgery, Qilu Hospital of Shandong University, West Wenhua Road No.107, Lixia District, Jinan, 250012 Shandong Province China; 2 Department of Cardiovascular Surgery, Linyi People’s Hospital Affiliated to Shandong University, Jiefang Street No. 27, Linyi, 276000 Shandong Province China

**Keywords:** Patent foramen ovale, Percutaneous closure, Occluder, Cryptogenic stroke, Migraine, Echocardiography, Transesophageal

## Abstract

**Background:**

Percutaneous closure of patent foramen ovale (PFO) is routinely performed using plain fluoroscopy in the catheter room. This method results in inevitable radiation damage, adverse effects of contrast agents on kidneys, and high cost. We performed PFO closure with a simplified and economical transesophageal echocardiography (TEE)-only guided approach in the operating room. This study aimed to investigate the feasibility, safety, and effectiveness of the percutaneous closure of PFO by only using TEE.

**Methods:**

We reviewed the medical records of patients who underwent percutaneous PFO closure at our center from December 2013 to December 2017. A total of 132 patients with PFO and cryptogenic strokes underwent PFO closure by using cardi-O-fix PFO device under TEE guidance. The participants comprised 64 and 68 male and female patients, respectively. The mean age and body weight of the patients were 39.40 ± 13.22 years old (12–68 years old) and 65.42 ± 9.70 kg (40–95 kg), respectively. All patients only received aspirin (3–5 mg/kg body weight, oral administration) for 6 months. Contrast-enhanced transthoracic echocardiography (c-TTE) with Valsalva maneuver was performed during follow-up, and questionnaire surveys were obtained at 3, 6, and 12 months after the procedure.

**Results:**

All (100%) patients were successfully closed. Follow-ups were conducted for 13 months to 48 months, with an average of 27 months. No severe complications were found during the follow-up period. Paroxysmal atrial fibrillation occurred in 4 patients within 3 months after the procedure. No recurrent stroke or death occurred in all patients during the follow-up period. Transient ischemic attack occurred in one patient 6 months after the procedure. Ten (7.6%) patients had a right-to-left shunt, as demonstrated by c-TTE at 12 months of follow-up. Among the 57 patients suffering from migraine, significant relief or resolution was reported by 42 (73.7%) patients.

**Conclusion:**

TEE-only guided PFO closure was a safe, feasible, and effective method that did not require the use of X-rays and contrast agents.

## Background

Patent foramen ovale (PFO) has been implicated in the pathogenesis of cryptogenic stroke, arterial desaturation, decompression illness, and migraine [1]. Percutaneous PFO closure is an option for patients with a cryptogenic stroke caused by the paradoxical embolism through the PFO [2]. Among patients with PFO and cryptogenic stroke, closure has reduced recurrent stroke and has a statistically significant effect on the composite of stroke, transient ischemic attack (TIA), and death in adjusted analyses [3]. Percutaneous closure is the preferred treatment, because it results in a smaller wound and a lower rate of postprocedural complications than open heart surgery [4, 5]. In most hospitals, the percutaneous procedures are performed using plain fluoroscopy in the catheter room. This procedure has some disadvantages, including the following: the inevitable radiation damage to patients and operators; the adverse effects of contrast agents on kidneys; and high cost. Moreover, when serious complications occur, e.g., occlude falling off or iatrogenic injury leading to cardiac tamponade, thoracotomy or extracorporeal circulation cannot be immediately performed [6–8]. To avoid the abovementioned disadvantages, transesophageal echocardiography (TEE) with clear sound window is used for patients. We proposed a TEE-guided transcatheter PFO closure approach.

Since the beginning of our program of the percutaneous closure of PFO, we used a simplified and economical TEE-only guided approach that is feasible, safe, and effective. We retrospectively reviewed cases in our hospital to evaluate this approach.

## Methods

### Patients

This study was approved by the Ethics Committee of Linyi people’s hospital affiliated to Shandong university; and for every procedure, an informed written consent was obtained from each patient. From December 2013 to December 2017, 132 patients with PFO and cryptogenic strokes underwent TEE-guided PFO closure. All patients experienced cryptogenic ischemic stroke and had PFO with a right-to-left shunt (RLS) prior to the percutaneous closure. Patients with PFO were diagnosed according to the results of transcranial Doppler (TCD) foaming test with Valsalva maneuver and transthoracic echocardiography (TTE). If TTE failed to identify PFO, TEE was performed. Careful examination was performed to assess the sizes and morphology of PFOs. No patient had contraindicated disease for PFO closure. RLS through a PFO was assessed via c-TTE with Valsalva maneuver. The classification of shunt size was based on the maximum number of microbubbles seen in the left atrium in any single frame during the first three cardiac cycles after detection in the right atrium. The presence of 0, 1–10, 11–30, and > 30 microbubbles were classified as no shunt, small, moderate, and large, respectively.

### Procedure

**Fig. 1:**
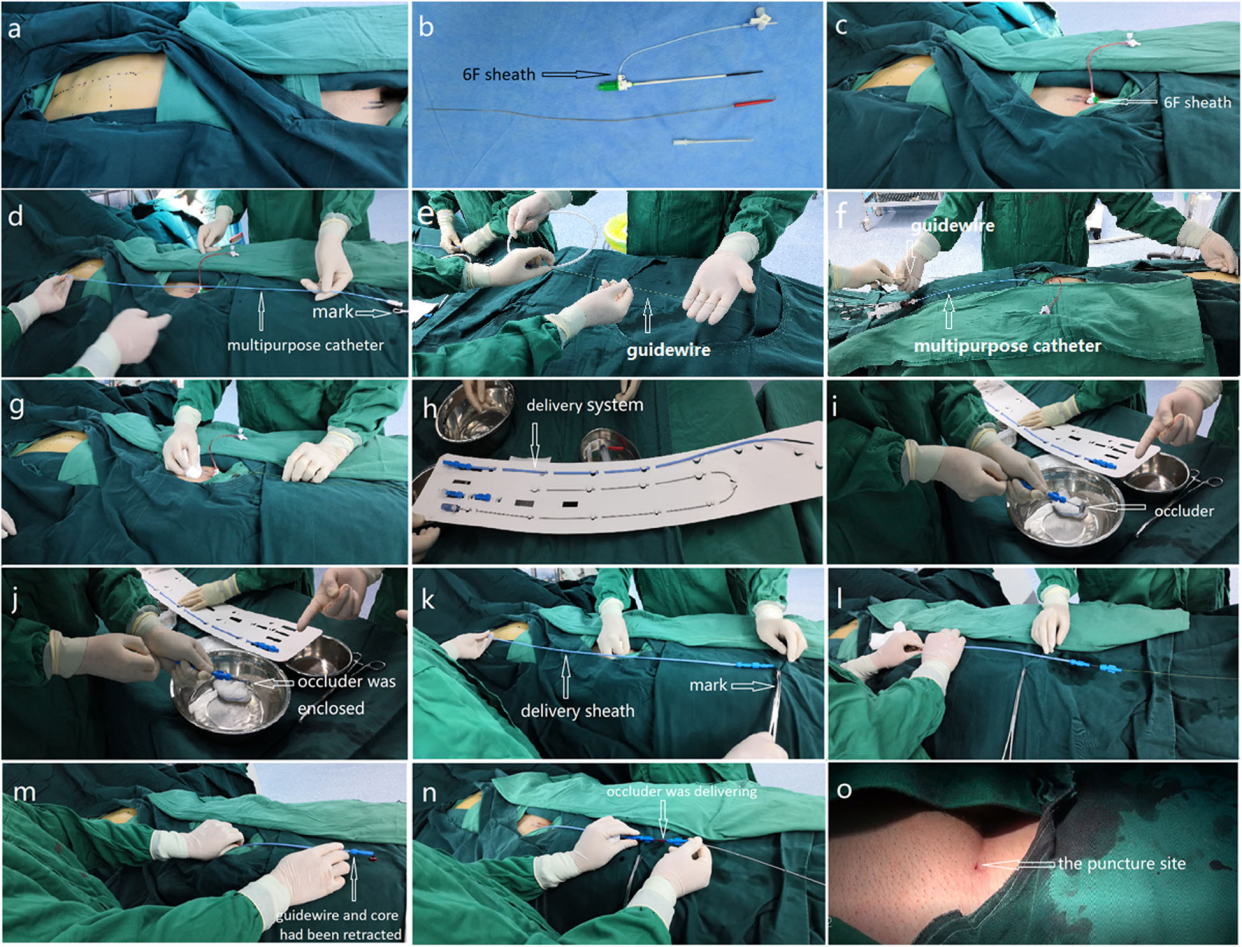
Procedure of a PFO closure under TEE guidance. **a** The closure was performed in the operating room. A 6F sheath was introduced after the vein was punctured (**b, c**). The distance from the right parasternal third intercostal space to the puncture site was measured and marked before the procedure for catheter insertion (**d**). This distance was also measured for the following insertion procedure for the super stiff guide wire and delivery sheath (**f, k**).** e** The 0.035'' super stiff guidewire. **g** The MP  was removed while the guidewire remained fixed in the left atrium. **h** The delivery system.** i **The occluder.** j** The occulder was enclosed. **l** The delivery sheath was inserted into the left atrium along the guide wire. **m** The guidewire and core had been retracted. **n** The device was fed along this pathway to the left atrium for PFO closure.** o** The puncture site

**Fig. 2:**
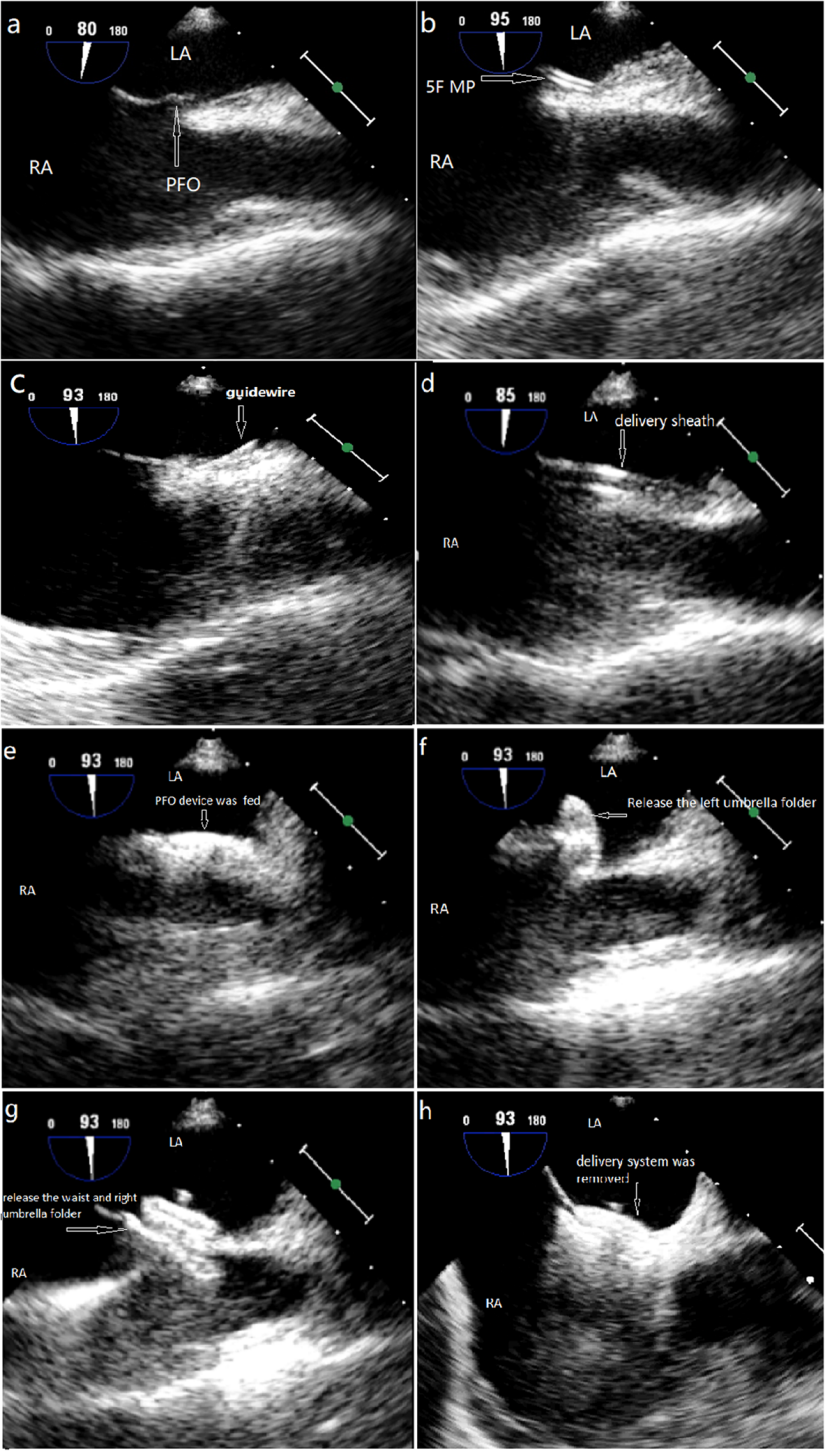
Percutaneous closure of a PFO under TEE guidance. **a** The location of the PFO was determined by TEE during the procedure. A 6Fr multi-purpose catheter (**b)**, guide wire (**c)**, and delivery sheath (**d)** were inserted through the PFO. **e** The device was fed. **f** The left umbrella folder was released. **g** The waist and right umbrella folder were released. **h** The PFO was closed. RA, right atrium; LA, left atrium

Patients received general anesthesia with endotracheal intubation. The TEE (Philips IE-33) probe was inserted. All procedures were performed by the same cardiac surgeon under TEE guidance in the operating room (Figs. 1 and 2). PFO occluder system (Starway Medical Technology, Inc., Beijing, China) was selected. In general, we used the right femoral vein as the catheter path, and a 6F sheath was introduced after the vein was punctured (Fig. 1c). We intravenously administered 100 ul/kg of heparin before the procedure. Before the procedure, the distance from the right parasternal third intercostal space to the puncture site was roughly measured and marked for catheter insertion (Fig. 1d). This distance was also important for the subsequent insertion procedure for the super stiff guide wire and delivery sheath, because the length of their entry needed to be controlled to avoid damaging heart tissue (Fig. 1d, f, and k). We inserted a 6F or 5F multipurpose catheter (MP), selected the appropriate tomographic sections of TEE to show the superior and inferior vena cava and the atrial septum, and guided the catheter to pass through the PFO to the left atrium (Fig. 2a and b). Rotating the catheter in this process several times was necessary to adjust the position of the tip of the catheter, thereby pointing it to the direction of the tunnel and passing it through the foramen ovale. The catheter was fixed, and a 0.035″ super stiff guidewire was inserted into the MP and beyond the top of the catheter (Fig. 2c). The catheter and sheath were removed while the guidewire remained fixed in the left atrium. A 8F to 12F delivery sheath was then selected according to the size of the occluder and inserted into the left atrium along the guide wire (Figs. 1l and 2d). We observed the top of the sheath, created a pathway to deliver the occlusion device, and retracted the wire and core (Fig. 1m). An 18–35 mm cardi-O-fix PFO device was fed along this pathway to the left atrium for PFO closure under TEE guidance (Figs. 1n and 2e). We released the left umbrella folder and withdrew the delivery system to close the atrial septum and retracted the sheath to release the waist and right umbrella folder of the occlusion device (Fig. 2f and g). After successfully implanting the device, suitable views were used to assess the position of the occluder and the possible effects on surrounding structures. Color doppler flow were used to observe atrioventricular valve function, coronary sinus return, systemic/pulmonary venous return, and residual shunting. After the occluder was securely implanted, the delivery system was removed, and pressure was applied at the puncture site for hemostasis. Endotracheal intubation was removed when the patient awoke in the operating room or intensive care unit.

### Follow-up

One day after the procedure, TTE and chest X-rays were performed to check for device position and any residual shunt or other peri-procedural complications. Electrocardiograms were checked for arrhythmia. All patients were discharged at 24–48 h with only aspirin (3–5 mg/kg body weight, oral administration) for 6 months. At 3, 6, and 12 months, all patients underwent TTE and c-TTE with Valsalva maneuver again. The above examinations were performed by the same sonographer who participated in the procedures. Further annual clinical follow-up was scheduled for all patients. The main concern was whether new arrhythmia, recurrent stroke, aortic erosion, and other complications occurred. Headache impact test-6 (HIT-6) scores were used to evaluate migraine symptomatology and compare the pre- and post-procedural headache severities of patients with migraine [9]. The questionnaire for verifying stroke-free status (QVSFS) was used to detect potential stroke or TIA [10]. If a new TIA or stroke was suspected, then a brain imaging examination was performed. For patients with palpitations, chest pain, or other symptoms, dynamic electrocardiogram examination needed to be performed.

### Statistical analyses

Statistical analysis was performed using the SPSS 13.0 software (SPSS Inc., Chicago, IL). Continuous data are presented as mean ± standard deviation, and categorial variables are presented as number and percentage. Postoperative and preoperative HIT-6 scores were compared using paired t-test. A two-tailed *P* < 0.05 was considered significant.

## Results

1. From December 2013 to December 2017, 132 patients with PFO and cryptogenic stroke underwent TEE-guided PFO closure. The characteristics, data, and procedural results of the patients with PFO are presented in Table 1,Table 2,Table 3and Figs. 1 and 2. There were 64 male patients and 68 female patients who participated. The mean age and body weight of the patients were 39.40 ± 13.22 years old (12–68 years old) and 65.42 ± 9.70 kg (40–95 kg), respectively. Seventy-nine cases (59.8%) of simple type of PFO (overlap length < 8 mm between the two septum, no atrial septal aneurysm, and no overlong Eustachian valve or Chiari network) and 53 (40.2%) cases of complex type of PFO were found. Fifty-seven (43.2%) patients with PFO suffered from migraine. The mean procedural time (from percutaneous femoral vein puncture to the removal of the sheath) was 29.76 ± 9.67 min (12–73 min). The mean hospitalization costs were 4124.0 ± 379.2 USD (3812.4–5130.3 USD). The mean postoperative hospital stay was 1.57 ± 0.61 days (1–3 days). The selection of the specific cardi-O-fix occluder was detailed in Table 1. The 18/18, 18/25, 25/25, 25/35, and 30/30 mm occluder devices were implanted in 23, 25, 72, 3, and 9 patients, respectively. Finally, the mean sizes of the occlusion device were 22.8 ± 3.8 and 24.3 ± 3.5 mm for the right and left disks, respectively.
2. Follow-ups were conducted for 13 months to 48 months (27 months on average). TIA occurred in one patient 6 months after procedure, and a small amount of RLS was found during post-procedural follow-up. No recurrent stroke or death occurred in all patients during the follow-up period. The rates of complete occlusion at 3, 6, and 12 months after procedural are shown in Table 3. Ten (7.6%) patients had RLS, as demonstrated by the c-TTE at 12 months of follow-up. Among them, four (3.1%) patients were moderate-to-large RLS. Considering migraine symptoms among the 57 patients with PFO, significant relief or resolution was reported by 42 (73.7%) patients 12 months after closure. The pre- and post-procedural HIT-6 scores for patients with migraine are shown in Table 2. The HIT-6 scores of 3, 6, and 12 months after closure were all significantly lower than those before closure (*P* < 0. 05).
3. The percutaneous transcatheter closure of TEE-guided PFO was performed successfully in all patients. We repeated a procedure to upsize the first selected device in three patients. This device had initially failed to stabilize in place. With the application of MP, PFO can easily be passed in most patients. Irregularly shaped PFOs, which were encountered in 13 (9.8%) in our series, were difficult to cross. We had to rotate the catheter back and forth several times in the fovea ovale area of nine patients to adjust the tip of the multi-purpose catheter before passing through the PFO. Straight-tip loach guidewire was used to pass the PFO through in three patients. Transseptal puncture was performed in only one patient, and a large RLS was found in this patient at 12 months of follow-up. Complications such as displacement of occlude, pericardial effusion, aortic erosion, infective endocarditis, occluder thrombosis, hemolysis, and femoral arteriovenous fistula were found during the follow-up period. Paroxysmal atrial fibrillation occurred in four patients within 3 months after the procedure. All four patients reverted to sinus rhythm after anti-arrhythmia treatment within 1 month after the onset.

**Table 1:** Characteristics, procedural features and outcomes of 132 consecutive patients submitted to Device Closure

Parameter	PFO
No of patients	132
Men/Women	64/68
Age (years) (range)	39.40 ± 13.22 (12–68)
Diameter of the PFO (mm)	2.37 ± 0.95
Migraine	57 (43.2%)
IASA	45 (34.1%)
Eustachian valve	15 (11.4%)
Procedural time (min)	29.76 ± 9.67
Successful closure	132 (100%)
Complications (Paroxysmal atrial fibrillation)	4 (3.0%)
Follow-up (months)	27.0 ± 9.1
Stroke recurrence	0
Migraine improvement	42/57 (73.7%)
Selection of the occluder	
18/18 mm	23 (17.4%)
18/25 mm	25 (18.9%)
25/25 mm	72 (54.5%)
25/35 mm	3 (2.3%)
30/30 mm	9 (6.8%)
right disk diameter (mm)	24.3 ± 3.5
left disk diameter (mm)	22.8 ± 3.8

*IASA* Interatrial septal aneurysm, *PFO* Patent foramen ovale

**Table 2:** Comparison of HIT-6 score before and after closure

Time	HIT-6	t	P
Preoperative	59.51 ± 4.52		
3 months	53.05 ± 6.05 △	9.012	< 0.05
6 months	51.09 ± 6.38 △	10.176	< 0.05
12 months	49.96 ± 7.13 △	9.948	< 0.05

*HIT-6* Headache Impact Test-6, *△*: *P* < 0.05 compared with the preoperative value.

**Table 3:** Outcomes of c-TTE before and after procedure

classification of shunt size	Preoperative	3 months	6 months	12 months
no shunt	0 (0%)	91 (68.9%)	111 (84.1%)	122 (92.4%)
small	0 (0%)	23 (17.4%)	11 (8.3%)	6 (4.5%)
moderate	25 (18.9%)	10 (7.6%)	6 (4.5%)	3 (2.3%)
large	107 (81.1%)	6 (6.1%)	4 (3.0%)	1 (0.8%)

*c-TTE* Contrast-enhanced transthoracic echocardiography, the presence of 0, 1–10, 11–30, and > 30 microbubbles were classified as no shunt and small, moderate, and large shunts, respectively.

## Discussion

The foramen ovale is a potential door capable of opening from the right to left atrium. Approximately 20 to 25% of the population has PFO. In young stroke patients, the prevalence reached 45% [11]. Patients with PFO are several times more likely to have stroke, migraine, peripheral arterial embolism, decompression sickness, and other risks than the normal population. The pathogenicity of PFO attracted the attention of numerous experts and scholars, and PFO closure has been explored to prevent recurrent stroke events, treat migraine, and platypnea-orthodeoxia [12]. Although the efficacy of PFO closure for cryptogenic stroke has been controversial, numerous experimental studies showed that PFO closure is superior to medical therapy in terms of preventing further stroke. Several recent meta-analyses found that PFO closure showed a marked benefit in preventing recurrent stroke/TIA with increased incidence of atrial fibrillation, and patients with moderate-to-large shunts or aged less than 60 years old had benefitted from interventional closure. Guidelines must be updated to reflect this procedure [13–15].

In most centers, fluoroscopy-guided approach has been generally used for the percutaneous closure of PFO, and TEE is only used for pre- or intra-procedural evaluation. However, X-rays emitted by fluoroscopy negatively affect the health of patients and operators. The contrast agents used in the process of digital subtraction angiography may also adversely affect the health of patients. The TEE-only guided occlusion of several simple congenital heart diseases, such as atrial septal defect (ASD), ventricular septal defect, and patent ductus arteriosus, is safe and effective [16–18]. PFO occlusion is similar to ASD occlusion, but PFO occlusion has its particularity. The main difficulty of PFO occlusion is how the catheter passes through the PFO channel. TEE feasibly provides guidance throughout the entire PFO closure procedure. TEE can clearly show the location of the catheter and guide the catheter through PFO to establish the path during the occlusion process. Accurately understanding the morphology of PFO in patients to improve the success rate of procedures and reduce complications is necessary for operators. Meanwhile, echocardiography is better than radiation guidance in assessing anatomy and hemodynamics [19]. TEE can observe the following in detail: the sizes of inlet and outlet of PFO, the tunnel length of PFO, the thickness of secondary septum, whether interatrial septal aneurysm was combined and its sizes, and whether abnormal eustachian valve and other abnormalities exist. TEE can also be used to monitor the whole process of occluder release, especially when determining whether or not the occluder was correctly placed, finding whether residual shunts exist, whether it affected the heart valves or coronary sinus, and whether aortic erosion occurs. The use of TEE also allowed for occluder replacement if the occluder was incorrectly positioned. After completely releasing the occluder, TEE was also applied to reevaluate the effect of the occlusion. The main advantages of this type of approach is the avoidance of X-ray radiation and contrast agent application. Moreover, incision is not necessary. All of our procedures were performed in the operating room, and the disinfected areas included the chest and groin to avoid delay in transfer or re-anesthesia of the patients in the event of severe complications (detachment of occluder or pericardial tamponade) due to operational failure. The procedure can be immediately converted to thoracotomy. In our procedures, the patients usually need general anesthesia with endotracheal intubation, which is a fly in the ointment. Professor Pan XB from Fuwai hospital in Beijing had successfully performed the ASD closure for some patients by only using TTE [8]. We may perform PFO closure for some suitable patients whose pictures are sufficiently clear by only using TTE under local anesthesia. However, TTE cannot provide a picture as clear as that produced by TEE and can be highly inaccurate in overweight, barrel-chested patients [7].

PFO is closely associated with migraines, but the underlying mechanism of PFO-induced migraine remains unclear. One hypothesis states that some specific metabolites such as serotonin and endothelial vasoconstrictor peptide, or subclinical emboli that enter the cerebral circulation system through PFO cause the migraine [20, 21]. A clinical meta-analysis shows that patients with PFO are 2.5 times more likely to suffer from migraines and 3.4 times more likely to suffer from migraines with aura [22]. The 25% incidence of PFO in the general population increased to 50% in the migraine population. Whether or not the PFO closure is effective in treating migraines remains controversial. Several randomized studies have failed to confirm that the improvement in migraine is due to the closure of the foramen ovale. Some retrospective and observational studies have reported the high rates of migraine relief (ranging from 50 to 80%) after PFO closure [2, 12]. In the present study, migraine relief was reported in 73.7% among the 57 patients after the closure procedure. The proportion of patients with a score of more than 60 before the closure procedure was as high as 57.9%, whereas a score of more than 60 indicated that headache significantly affected the family, work, study, or social activities of the patients, and their quality of life has significantly decreased. Twelve months after occlusion, nearly 50% of the patients scored 15 points lower than the preoperative scores, and the total score was less than 50 points, thereby indicating that migraine symptoms disappeared or significantly decreased in most patients.

PFO occlusion showed good efficacy despite the lack of randomized control group. All PFOs (100%) were successfully closed using the cardi-O-fix PFO occluder. During long-term follow-up of 27.0 ± 9.1 months, recurrent stroke was not encountered, and severe complications were not detected. Paroxysmal atrial fibrillation occurred in only four (3%) patients after the procedure. However, although no blood flow signal between the atriums was observed by echocardiography after PFO closure, the residual right-left shunt in 39 (31.1%), 21 (15.9%), and 10 (7.6%) of 132 patients who underwent reexamination of foaming test at 3, 6, and 12, months after the procedure was observed, respectively. The high incidence of residual shunt in the follow-up period of this study may be due to the use of c-TTE for assessing RLS. In some other trials c-TEE was used for follow-up. TEE is a semi-traumatic and painful examination for patients, and completing Valsalva maneuver successfully during the process was difficult for some of them. Thus, the residual shunt may have been underestimated. In addition, the patient with a large residual RLS, as demonstrated by the c-TTE at 12 months of follow-up, was the one who had received transseptal puncture during the closure procedure. We assumed that the application of transseptal puncture may have contributed to the large residual shunt. Transseptal puncture technique may be an effective option for facilitating device closure after the failure of conventional approach, but an increased incidence of residual shunt was observed with this technique [23]. Comparative studies between Cardi-O-fix PFO occluder and other type of occluders have been rarely conducted. Perhaps the lack of effectiveness of the device was also a factor affecting the high rate of residual shunt. Moreover, other reasons for residual shunt after occlusion exists. Atrial-level shunt is not the sole source of paradoxical emboli. Pulmonary arteriovenous malformations, venous abnormalities, including persistent left-sided superior vena cava to the left atrium, or an unroofed coronary sinus may also cause paradoxical embolization. Positive results on bubble testing after PFO closure are not uncommon; important anatomic lesions may coexist and remain a potential risk factor for recurrent paradoxical embolization [24]. In the present study, the possibility of pulmonary vascular malformations in the four (3.1%) patients with a moderate-to-large residual RLS at 12 months of follow-up was ruled out by computed tomographic pulmonary angiography. The four patients were asked to take aspirin (3–5 mg/kg body weight) orally to prevent the recurrence of CS and to undergo a review c-TTE with Valsalva maneuver annually. If residual RLS drops to a small shunt, the aspirin treatment can be discontinued. If residual RLS does not reduce and neurology embolism event recurs, then secondary percutaneous intervention or surgical closure is recommended.

### Study limitations

This study was not a randomized controlled trial. This study was a documentation of our experience with a relatively small series of patients, and the follow-up period was only 13 months to 48 months. Further studies are required to establish long-term results in a larger patient population.

## Conclusion

In summary, TEE-only guided PFO closure allowed the avoidance of the use of X-rays and contrast agents and provided a safe, feasible, and effective method for the closure of PFO. The percutaneous transcatheter closure of PFO was performed successfully in all patients. According to the 12-month c-TTE follow-up study, more than 90% of patients did not have residual shunt. Mean long-term follow-up for > 2 years did not show recurrence of stroke. More than 70% of patients with migraine experience felt relieved after the closure procedure.

## Data Availability

Please contact author for data requests.

## Abbreviations

ASD: Atrial septal defect
c-TCD: Contrast-enhanced transcranial Doppler
c-TTE: Contrast-enhanced transthoracic echocardiography
DSA: Digital subtraction angiography
HIT-6: Headache Impact Test-6
IASA: Interatrial septal aneurysm
MP: Multipurpose catheter
PFO: Patent foramen ovale
QVSFS: Questionnaire for Verifying Stroke-Free Status
RLS: Right-to-left shunt
TCD: Transcranial Doppler
TEE: Transesophageal echocardiography
TIA: Transient ischemic attack
TTE: Transthoracic echocardiography
